# Prevalence of Human African Trypanosomiasis in the Democratic Republic of the Congo

**DOI:** 10.1371/journal.pntd.0001246

**Published:** 2011-08-02

**Authors:** Dieudonne Mumba, Elaine Bohorquez, Jane Messina, Victor Kande, Steven M. Taylor, Antoinette K. Tshefu, Jeremie Muwonga, Melchior M. Kashamuka, Michael Emch, Richard Tidwell, Philippe Büscher, Steven R. Meshnick

**Affiliations:** 1 Institut National de Recherche Biomédicale, Kinshasa, Democratic Republic of the Congo; 2 Department of Epidemiology, University of North Carolina Gillings School of Global Public Health, Chapel Hill, North Carolina, United States of America; 3 Department of Geography, University of North Carolina, Chapel Hill, North Carolina, United States of America; 4 Programme National de Lutte contre la Trypanosomiase Humaine Africaine, Kinshasa, Democratic Republic of the Congo; 5 Kinshasa School of Public Health, University of Kinshasa, Kinshasa, Democratic Republic of the Congo; 6 Programme National de Lutte contre la SIDA, Kinshasa, Democratic Republic of the Congo; 7 Department of Pathology and Laboratory Medicine, University of North Carolina School of Medicine, Chapel Hill, North Carolina, United States of America; 8 Department of Parasitology, Institute of Tropical Medicine, Antwerp, Belgium; International Centre of Insect Physiology and Ecology, Kenya

## Abstract

Human African Trypanosomiasis (HAT) is a major public health problem in the Democratic Republic of the Congo (DRC). Active and passive surveillance for HAT is conducted but may underestimate the true prevalence of the disease. We used ELISA to screen 7,769 leftover dried blood spots from a nationally representative population-based survey, the 2007 Demographic and Health Survey. 26 samples were positive by ELISA. Three of these were also positive by trypanolysis and/or PCR. From these data, we estimate that there were 18,592 people with HAT (95% confidence interval, 4,883–32,302) in the DRC in 2007, slightly more than twice as many as were reported.

## Author Summary

Because of weak health surveillance infrastructures in poor countries, estimates of the burdens (numbers of infections) of many tropical diseases may be inaccurate. In particular, current estimates for the global burden of Human African Trypanosomiasis (Sleeping Sickness, HAT) vary widely. Most of the reported HAT cases occur in the Democratic Republic of the Congo, where many barriers to surveillance exist. The best way to generate accurate burden estimates is to use a survey sampled to be representative of the general population. Demographic and Health Surveys (DHS) are a widely used tool to obtain nationally representative health data and have been conducted hundreds of times in developing countries, In this report, we use samples from the 2007 Democratic Republic of the Congo DHS to estimate the burden of HAT. ELISA tests were conducted on 7,769 leftover dried blood spots followed by confirmatory trypanolysis and PCR tests. Our data suggest that there are approximately 18,592 cases of Human African Trypanosomiasis (Sleeping Sickness) in the DRC, close to WHO estimates.

## Introduction

Human African trypanosomiasis (HAT) has been reported in most of sub-Saharan Africa as well as in travelers to the region [1]. Currently, the global prevalence of the disease is uncertain. From 2006 to 2008, there were 7200–8200 reported cases of HAT per year [2]. However, since HAT occurs in remote areas with poor health infrastructures, under-reporting is likely. Thus, estimates of the global burden have been as high as 300,000 [3].

Nearly two-thirds of all reported HAT cases are from the Democratic Republic of the Congo (DRC). However, the DRC is a huge country (2.3 million km^2^) with poor infrastructure and only 2,794 km of paved roads (https://www.cia.gov/library/publications/the-world-factbook/geos/cg.html). Only 19% of the presumed at-risk population was screened in 2003 [4]. High prevalence of HAT was found recently in surveillance “blind spots” both in the DRC and elsewhere [5]. Thus, the true number of HAT cases could be much higher than the numbers reported to the World Health Organization (WHO).

The problem of over- and underestimating the prevalence of diseases is not unique for HAT. One approach to obtaining accurate assessments of disease prevalence is through nationally representative health surveys [6]. Demographic and Health Surveys (DHS) are a widely used method to obtain nationally representative data and have been conducted hundreds of times in developing countries (http://www.measuredhs.com/). Since 2001, many DHS have included dried blood spots from participants to be used for a more accurate assessment of HIV seroprevalence. Seroprevalences determined this way are not subject to selection biases and are often quite different from results obtained using sentinel populations such as those who attend antenatal care clinics. Recently, using these new data, the WHO revised its estimates of the global prevalence of HIV [7].

In this study, we attempt to obtain a population-based estimate of HAT prevalence in the DRC. To accomplish this, we screened 7,769 leftover dried blood spots from the 2007 DRC DHS.

## Methods

### Study subjects

**Figure 1:**
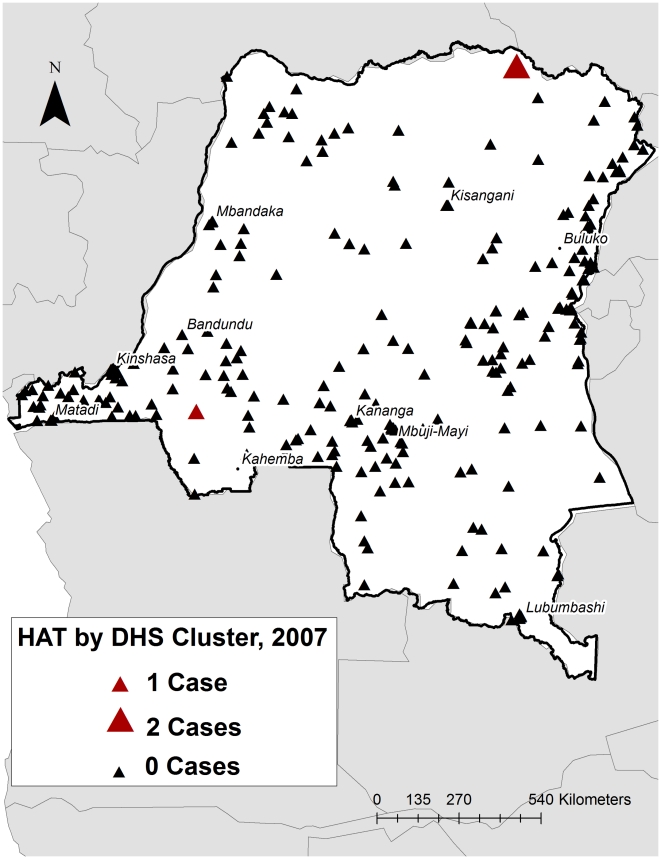
Map of the 300 sites from which dried blood spots were obtained and where the positive cases were detected.

The survey methodology was described previously [8], [9]. Briefly, a 2-stage stratified cluster design based on a national survey was used to generate nationally representative data on population, health and social indices. Nine thousand households from 300 randomly selected population-representative geographic clusters (Fig. 1), were selected for inclusion; all women aged 15 to 49 years within these households were surveyed, and, in half of the households, men aged 15 to 59 were surveyed. All men and half of the women were consented for collection of blood spots. The specimens were originally collected for the determination of HIV seroprevalence and were deidentified before we received them. Our study received ethical approval from the Institutional Review Boards of the Kinshasa School of Public Health and the University of North Carolina.

### HAT ELISA

The ELISA for *T.b. gambiense* was performed as described by Hasker et al. with some modifications [10]. From each dried blood spot, two 5 mm diameter disks were punched and eluted in 1 ml elution buffer. The eluted fraction was separated from the disks and assayed in duplicate both in the presence and absence of antigen consisting of a mixture of purified *T.b. gambiense* LiTat 1.3, and LiTat 1.5 each at a concentration of 1 µg/ml, coated at 150 µl per well in microtitre plates(Maxisorp, Nunc). ELISA results were expressed as percent positivity relative to a positive control serum run in each plate. A result was considered positive if ≥ 45%.

### Trypanolysis

Immune trypanolysis was performed according to Van Meirvenne et al. [11] with cloned live populations of *T.b. gambiense* Variable Antigen Types (VATs) LiTat1.3 and LiTat 1.5 and one *T.b .rhodesiense* VAT ETat 1.2R. The test was adapted for testing blood impregnated filter paper according to Holland et al. [12]. Briefly, from each dried blood spot, a 6 mm diameter disk was punched and placed in a well of a flat-bottom microtitre plate containing 20 µl of guinea pig serum (complement source). The plate was covered with a lid and put at 4°C on a microtitre plate shaker for elution. After one hour, 10 µl of a 10^7^ trypanosomes/ml suspension in guinea pig serum were added to each well, leaving the filter paper disks in place. The plate was incubated at ambient temperature and shaken for 30, 60 and 90 minutes. After 90 minutes, the suspension in each well was examined under the microscope (25×10 magnification) for living trypanosomes. Trypanolysis was considered positive when >50% of the trypanosomes were lysed. *T.b. rhodesiense* VAT ETat 1.2R was used as a control for the absence of non-specific trypanolytic activity of the test specimens.

### HAT PCR

Genomic DNA (gDNA) was extracted from dried blood spots using the invitrogen Purelink 96 kit (invitrogen, Carlsbad, CA) as described (Taylor et al, submitted). A TaqMan®-MGB real-time PCR assay targeting the 177 bp satellite repeat was developed, modeled after a published SYBR Green method [13]. Applied Biosystems Primer Express Software 3.0 was used to design real-time PCR primers and FAM-labeled probe to amplify this region (Table 1). Primer specificity was evaluated through a BLAST search of the human genome.

**Table 1:** Sequences and properties of real-time PCR primers and probe for 177 bp satellite repeat.

Name	Sequence	%GC	Tm (°C)
F Primer	GCGCAGTTAACGCTATTATACACA	41.7	59.3
R Primer	TTAAACACTAAAGAACAGCGTTGC	37.5	57.6
Probe	FAM-CAAGTGTGCAACATTAAATACAAGTG-MGB	35	72

After optimizing the assay, each real-time PCR reaction contained 1 µg of gDNA, ABI's TaqMan® MasterMix (10×), 100 nM probe, 300 nM of each primer, and nuclease-free water to reach a total reaction volume of 25 µL. The real-time PCR reactions were carried out using an ABI PRISM® 7000 Sequence Detection System (Applied Biosystems, Inc., Foster City, CA, USA) under the following conditions: 50°C for 2 minutes, 95°C for 10 min., and 45 cycles of 95°C for 15 seconds and 60°C for 1 min. Human gDNA extracted from whole blood and nuclease-free water were used as negative controls. *T. b. brucei* DNA at 0.1 ng/µL was used as the positive control. All samples and controls were run in duplicate or triplicate.

The sensitivity and specificity of this PCR assay was determined on human samples previously obtained [14]. Informed consent from Congolese, Ugandan and Dutch patients was obtained. The study was approved by the Ethical Committee of the University of Antwerp (reference number: B3002006603). Blood was collected from 59 patients, of whom 33 had peripheral parasites and 26 had CSF parasites only. Control blood was collected from 50 healthy individuals in Uganda, the DRC, and the Netherlands.

### Statistical analyses

HAT prevalence was determined by calculating the proportion of the 7,769 samples which were positive using the sampling weights of the DHS survey (in this case the three positive cases were weighted as2.3). This proportion was then multiplied by the total mid-year population of the DRC in 2007, estimated by the Population Reference Bureau to be 62.6 million [15]. The confidence interval was computed using the standard error of the percentage calculated by SAS Proc Surveyfreq, which accounts for sampling weights and clustering of the samples tested.

## Results and Discussion

**Figure 2:**
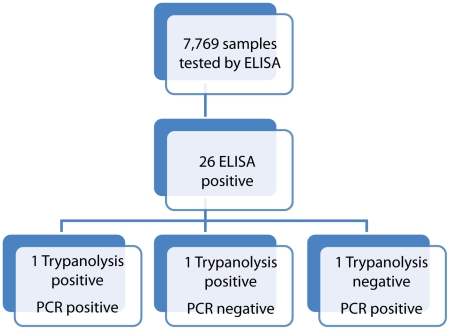
Flow chart showing processing of samples.

A total of 7,769 samples were tested by ELISA (Fig. 2). Of these samples, 26 specimens in 23 sites were found to be positive (data not shown).

For PCR confirmation of ELISA-positive specimens we developed a Taqman real-time PCR assay to improve specificity. Using previously collected samples (not from the DRC DHS set), this PCR assay was positive in 33% (10/31) of the subjects with known microscopy-confirmed HAT in peripheral blood samples and none of the uninfected patients (0/17). Thus, this assay had low sensitivity but very high specificity.

All 26 ELISA-positive samples were tested by both trypanolysis and PCR (Fig. 2). Two subjects were positive by trypanolysis with 100% lysis on both LiTat 1.3 and LiTat 1.5 VAT. One of these trypanolysis-positive specimens was also positive by PCR. In addition, one trypanolysis-negative subject was positive by PCR, suggesting a recent infection that had not yet elicited anti-trypanosomal antibodies.

The other 23 ELISA positive samples which were negative by PCR and trypanolysis are likely to be false positives. This false positive rate (23/7766) translates to a very high ELISA test specificity (99.7% with 95% CI: 99.5–99.9). However, given the low prevalence of the disease, the ELISA's positive predictive value is only 11.5% (95% CI: 4.0–28.9).

All 3 trypanolysis and/or PCR-positive subjects were male and were HIV-seronegative. Two were co-infected with *P. falciparum* [8]. These 3 cases were found in two sites, both of which are in known endemic regions (Fig. 1).

The overall prevalence of HAT in the DRC was calculated using standard sampling weights and found to be 29.7 cases/100,000 persons. Assuming a total population of 62.6 million, this leads to an estimated 18,592 people with HAT (95% confidence interval, 4,883–32,302) in the DRC in 2007.

In 2007, the National Trypanosomiasis Control Program reported 8,162 cases of HAT. Our results suggest that 56% of actual HAT cases were not detected and therefore not reported [2]. This is very close to estimates of underreporting used by the WHO (65–75%) [16].

The estimates obtained here are subject to several limitations. First, none of the tests are completely sensitive, so cases of HAT infection could have been missed. Second, HAT is a highly clustered disease, and it is possible that specific small geographic regions with high HAT prevalences were not accurately sampled. Both limitations would have led to understimates of the prevalence of the disease. Nevertheless, our results provide the first nationally representative population-based data on the prevalence of this disease and confirm WHO estimates for under-reporting. This study also confirm that population-based surveys are useful in determining the burden of infectious diseases.

## Supporting Information

Checklist S1 **STROBE checklist.**
(DOCX)
