# Supplementary material for: Prevalence of Human African Trypanosomiasis in the Democratic Republic of the Congo
Source: PLoS Negl Trop Dis. 2011 Aug 2;5(8):e1246. doi: 10.1371/journal.pntd.0001246 (PMC3149009; doi:10.1371/journal.pntd.0001246)
Supplement: Checklist S1 — STROBE checklist. (DOCX) [file pntd.0001246.s001.docx]

STROBE CHECKLIST

| Item |  |
| --- | --- |
| 1 | Yes |
| 2 | Intro, pars. 2 and 3. |
| 3 | Last paragraph of intro. |
| 4 | First paragraph of methods |
| 5 | First paragraph of methods |
| 6 | First paragraph of methods |
| 7 | 2^nd^ and 3^rd^ paragraphs of methods |
| 8 | 2^nd^ and 3^rd^ paragraphs of methods |
| 9 | Last paragraph of Results and Discussion |
| 10 | First paragraph of methods |
| 11 | Last paragraph of methods |
| 12 | Last paragraph of methods |
| 13 | Figure 2 |
| 14 | Not relevant. Fully described in refs 8 and 9 |
| 15 | Paragraphs 6 and 7 of Results and Discussion |
| 16 | Paragraph 6 of Results and Discussion |
| 17 | None |
| 18 | Paragraphs 6 and 7 of Results and Discussion |
| 19 | Last paragraph |
| 20 | Last paragraph |
| 21 | Last paragraph |
| 22 | Removed from MS as directed in email |
